# The Gly2019Ser mutation in *LRRK2 *is not fully penetrant in familial Parkinson's disease: the GenePD study

**DOI:** 10.1186/1741-7015-6-32

**Published:** 2008-11-05

**Authors:** Jeanne C Latourelle, Mei Sun, Mark F Lew, Oksana Suchowersky, Christine Klein, Lawrence I Golbe, Margery H Mark, John H Growdon, G Frederick Wooten, Ray L Watts, Mark Guttman, Brad A Racette, Joel S Perlmutter, Anwar Ahmed, Holly A Shill, Carlos Singer, Stefano Goldwurm, Gianni Pezzoli, Michela Zini, Marie H Saint-Hilaire, Audrey E Hendricks, Sally Williamson, Michael W Nagle, Jemma B Wilk, Tiffany Massood, Karen W Huskey, Jason M Laramie, Anita L DeStefano, Kenneth B Baker, Ilia Itin, Irene Litvan, Garth Nicholson, Alastair Corbett, Martha Nance, Edward Drasby, Stuart Isaacson, David J Burn, Patrick F Chinnery, Peter P Pramstaller, Jomana Al-hinti, Anette T Moller, Karen Ostergaard, Scott J Sherman, Richard Roxburgh, Barry Snow, John T Slevin, Franca Cambi, James F Gusella, Richard H Myers

**Affiliations:** 1 Department of Neurology, Boston University School of Medicine, Boston University, Boston, MA, USA; 2 Molecular Neurogenetics Unit, Center for Human Genetic Research, Massachusetts General Hospital, Harvard Medical School Boston, MA, USA; 3 Department of Neurology, University of Southern California, Los Angeles, CA, USA; 4 Departments of Clinical Neurosciences and Medical Genetics, University of Calgary, Calgary, Alberta, Canada; 5 Department of Neurology, Medical University of Lübeck, Lübeck, Germany; 6 Department of Neurology, University of Medicine and Dentistry of New Jersey-Robert Wood Johnson Medical School, New Brunswick, NJ, USA; 7 Department of Neurology, Massachusetts General Hospital, Harvard Medical School Boston, MA, USA; 8 Department of Neurology, University of Virginia Health System, Charlottesville, VA, USA; 9 Department of Neurology, University of Alabama at Birmingham, Birmingham, AL, USA; 10 Department of Medicine, University of Toronto, Toronto, Canada; 11 Department of Neurology, Washington University School of Medicine, Saint Louis, MO, USA; 12 Barrow Neurological Institute, Phoenix, AZ, USA; 13 Sun Health Research Institute, Sun City, AZ, USA; 14 Department of Neurology, University of Miami, Miami, FL, USA; 15 Parkinson Institute, Istituti Clinici di Perfezionamento, Milano, Italy; 16 Department of Biostatistics, Boston University School of Medicine, Boston University, Boston, MA, USA; 17 Departments of Neurology and Neuroscience, Cleveland Clinic Foundation, Cleveland, OH, USA; 18 Department of Neurology, University of Louisville School of Medicine, Louisville, KY, USA; 19 Neurology Department, University of Sydney ANZAC Research Institute, Concord Hospital, Sydney, Australia; 20 Struthers Parkinson's Center, Park Nicollet Clinic, Golden Valley, MN, USA; 21 Port City Neurology, Scarborough, ME, USA; 22 Parkinson's Disease and Movement Disorder Center of Boca Raton, Boca Raton, FL, USA; 23 Institute for Ageing and Health, Newcastle University, Newcastle upon Tyne, UK; 24 Regional Neurosciences Centre, Newcastle University, Newcastle upon Tyne, UK; 25 Department of Neurology, General Regional Hospital Bolzano, Bolzano, Italy; 26 Department of Neurology, University of Arkansas for Medical Sciences, AR, USA; 27 Department of Neurology, Aarhus University Hospital, Aarhus, Denmark; 28 Department of Neurology, University of Arizona, Tucson, AZ, USA; 29 Department of Neurology, Auckland City Hospital, Auckland, New Zealand; 30 Department of Neurology, University of Kentucky College of Medicine, Lexington, KY, USA

## Abstract

**Background:**

We report age-dependent penetrance estimates for leucine-rich repeat kinase 2 (*LRRK2*)-related Parkinson's disease (PD) in a large sample of familial PD. The most frequently seen *LRRK2 *mutation, Gly2019Ser (G2019S), is associated with approximately 5 to 6% of familial PD cases and 1 to 2% of idiopathic cases, making it the most common known genetic cause of PD. Studies of the penetrance of *LRRK2 *mutations have produced a wide range of estimates, possibly due to differences in study design and recruitment, including in particular differences between samples of familial PD versus sporadic PD.

**Methods:**

A sample, including 903 affected and 58 unaffected members from 509 families ascertained for having two or more PD-affected members, 126 randomly ascertained PD patients and 197 controls, was screened for five different *LRRK2 *mutations. Penetrance was estimated in families of *LRRK2 *carriers with consideration of the inherent bias towards increased penetrance in a familial sample.

**Results:**

Thirty-one out of 509 families with multiple cases of PD (6.1%) were found to have 58 *LRRK2 *mutation carriers (6.4%). Twenty-nine of the 31 families had G2019S mutations while two had R1441C mutations. No mutations were identified among controls or unaffected relatives of PD cases. Nine PD-affected relatives of G2019S carriers did not carry the *LRRK2 *mutation themselves. At the maximum observed age range of 90 to 94 years, the unbiased estimated penetrance was 67% for G2019S families, compared with a baseline PD risk of 17% seen in the non-*LRRK2*-related PD families.

**Conclusion:**

Lifetime penetrance of *LRRK2 *estimated in the unascertained relatives of multiplex PD families is greater than that reported in studies of sporadically ascertained *LRRK2 *cases, suggesting that inherited susceptibility factors may modify the penetrance of *LRRK2 *mutations. In addition, the presence of nine PD phenocopies in the *LRRK2 *families suggests that these susceptibility factors may also increase the risk of non-*LRRK2*-related PD. No differences in penetrance were found between men and women, suggesting that the factors that influence penetrance for *LRRK2 *carriers are independent of the factors which increase PD prevalence in men.

## Background

Parkinson's disease (PD) is a neurodegenerative disorder, affecting approximately 1.8% of individuals over the age of 65 [1]. Some cases of PD are due to known genetic or environmental factors but most are likely due to complex interactions among unidentified genes and environmental risk factors. These susceptibility factors may also play a role in the penetrance of known PD genes.

Mutations in the leucine-rich repeat kinase 2 gene (*LRRK2*) are the most common known genetic cause of PD. The most frequent *LRRK2 *mutation, G2019S, is estimated to be associated with 5% to 6% of familial PD and 1% to 2% of idiopathic cases in populations of European descent [2,3]. More than 20 additional rare mutations throughout the *LRRK2 *gene have been reported with varying frequency among different populations [2].

Studies of the G2019S mutation have reported a wide range of penetrance estimates. Early studies, performed in large families with multiple affected members, reported high lifetime penetrance for *LRRK2 *mutations, ranging from 70% [4] to 100% [5]. Subsequent studies of the age-dependent penetrance of G2019S mutations in families with multiple affected members have reported a range of penetrance estimates. Kachergus and colleagues [6] reported 17% penetrance at age 50 increasing to 85% at age 70 in 13 families segregating the G2019S mutation, while Lesage and colleagues [7] reported 33% penetrance at age 50, increasing to 100% at age 75 in 13 French and North African families ascertained for dominantly inherited PD.

More recent studies have screened ethnically diverse PD populations, who were not ascertained for familial history of the disease. These studies have generally reported lower lifetime penetrance estimates for the G2019S mutation, ranging from 22% to 32% [8-10], and decreased age-dependent penetrance ranging from 2% at age 50 to 33% at age 80 [11]. One possible explanation for the higher penetrance estimates reported among the family-based *LRRK2 *studies is an ascertainment bias created by more ready ascertainment of families with multiple affected members. Alternatively, genetic or environmental susceptibility factors shared by affected family members may also increase penetrance in some families.

In this study, we report age-dependent penetrance estimates for *LRRK2 *derived from a large sample of familial PD with consideration of the potential for bias inherent in a familial sample. Penetrance was estimated for families with either the G2019S or R1441C mutations, as well as for the G2019S mutation alone.

## Methods

### Subjects

Study participants were recruited by the GenePD study, an international multi-site study of the genetics of PD, using three ascertainment strategies: 1) PD-affected probands with a PD-affected sibling; 2) PD-affected probands with a PD-affected parent or offspring; and 3) PD-affected probands with another PD-affected relative such as a cousin, aunt or uncle. Both affected and unaffected relatives of the proband were recruited (Table 1). An additional series of 126 randomly ascertained PD cases was recruited through the BUMC/BU Neurology Associates clinic. Controls were recruited from the spouses or in-laws of PD patients at BUMC/BU Neurology Associates clinic and from four GenePD study site clinics. All participants gave informed consent and the study was approved by the Boston University Medical Center Institutional Review Board.

**Table 1:** Distribution by sex and mean ages of onset or enrollment for the idiopathic Parkinson's disease (PD), familial PD, unaffected PD relative and controls samples

	*N*	Men (%)	Women (%)	Mean age at onset or *enrollment
Randomly ascertained PD	126	81 (64%)	45 (36%)	56.3
Familial PD	903	501 (55%)	402 (45%)	60.4
Unaffected relatives	59	26 (44%)	33 (56%)	*58.7
Controls	197	95 (48%)	102 (52%)	*65.9

The diagnostic criteria for PD affection follow the United Kingdom Brain Bank criteria, and all probands were examined by a neurologist trained as a movement disorder specialist. When possible, the affection status of other family members was also confirmed by neurological examination. In cases where examination was not possible (for example, where the affected family member was deceased) PD affection status was determined through administration of a diagnostic questionnaire (adapted from Marder et al, estimated sensitivity = 95.5% and specificity = 96.2% [12]). Family history data was collected in a structured interview which collected information on the onset age and PD affection status for all first-degree relatives of the proband.

### Genotyping

Genotyping of the G2019S, R1441C, R1441H, Y1699C and I2020T mutations was performed using TaqMan technology implemented on the ABI PRISM^® ^7900 HT Sequence Detection system (Applied Biosystems: Foster City, CA) for 650 familial PD cases, 126 randomly ascertained PD cases, 125 controls and 30 unaffected relatives of familial PD cases. The mutations G2019S and R1441C, the only mutations observed in the initial sample size above, were typed in an additional 253 familial PD cases, 72 controls and 28 unaffected relatives of familial PD cases.

All variants identified by allelic discrimination assays were further confirmed by directly sequencing PCR products, which was performed by the DNA sequencing core at Massachusetts General Hospital using an ABI377/XL or an ABI3730 DNA analyzer. Forward and reverse primers were used with Applied Biosystems BigDye terminator v3.1 sequencing chemistry according to the manufacturer's protocol.

### Estimation of penetrance

Family history data for PD affection, age at onset and age at death were used to assess penetrance among the parents of sibships ascertained for at least two PD-affected siblings where at least one of these was determined to be a *LRRK2 *carrier. Only the parents from families ascertained as affected sibling pairs were used in this analysis, in order to avoid bias towards increased penetrance among those families ascertained as PD-affected parent-offspring pairs. Similarly, the siblings in these sibships themselves were not used in the assessment of penetrance, in order to avoid the bias of ascertainment of sibships with multiple affected members. The parents of PD-affected siblings were used to provide an unbiased sample in which to estimate penetrance because the likelihood of PD-affected siblings being ascertained into the study is independent of the affection status of their parents.

Penetrance estimates were made under the assumption that only one parent from each *LRRK2 *sibship was a carrier. Because nearly all the parents were deceased, it was not possible to determine which parent was the mutation carrier. Thus, one parent per sibship was selected as the *LRRK2*-carrying parent using the following criteria. In families where neither parent was PD-affected, one parent was selected at random. When one parent was PD-affected, selection was weighted to select the affected parent 98.5% of the time and the unaffected parent 1.5% of the time, to allow for the presence of PD phenocopies among affected parents. This weighting was derived from a conservative estimate of an underlying population prevalence of PD of 1.5% for the age range of the study sample. This selection process was repeated 1000 times, each time randomly selecting one *LRRK2 *carrier parent according to the above criteria, to produce a distribution of survival estimates for PD. Final penetrance estimates were the average of the 1000 penetrance estimates.

The PROC LIFETEST procedure in SAS v9.1 was used to produce the product-limit survival estimates for each event (for example PD onset) or censoring age. Penetrance was calculated for each age by subtracting the survival estimate from 1. The same estimation was performed in the set of non-*LRRK2 *carrying families ascertained as affected sibling pairs to permit a comparison of the age-dependent penetrance of *LRRK2 *families with the age-dependent risk of PD in families of unspecified etiology.

Differences in *LRRK2 *penetrance by sex were examined by testing the homogeneity of the survival curves for men and women using the Log-rank test for each of the 1000 randomly selected samples.

## Results

From the 903 familial PD cases studied, 58 *LRRK2 *mutation carriers (6.4%) were identified in 31 apparently unrelated families (Table 2). Four R1441C mutation carriers were identified in two families, while the remaining 54 carriers had G2019S mutations. Six G2019S mutations were identified in the 126 randomly ascertained PD cases (4.8%). No mutations were identified among the controls or unaffected relatives of PD cases. Nine PD-affected relatives of G2019S carriers did not carry the *LRRK2 *mutation themselves, suggesting that some multiplex families have multiple sources of PD and that PD phenocopies are found among *LRRK2 *families.

**Table 2:** Subjects are presented according to the method of ascertainment, with the number of unaffected relatives genotyped for *LRRK2 *mutations shown in parenthesis

Ascertainment method	Subjects (unaffected relatives)	Families	*LRRK2 *mutations (%)	*LRRK2 *families (%)
Total familial Parkinson's disease cases and families studied	903 (58)	509	58 (6.4%)	31 (6.1%)
• Affected siblings	730 (37)	401	47 (6.4%)	24 (6.0%)
• Affected parent-offspring	141 (20)	88	11 (7.8%)	7 (8.0%)
• Other affected relatives	32 (1)	20	0	0
				
Randomly ascertained PD	126	126	6 (4.8%)	6 (4.8%)
Controls	197	197	0	0

No controls or unaffected family members were determined to be *LRRK2 *carriers.

The majority of families in this study were recruited as affected sibling pairs (401 of 509 families or 79%), and 6% of these were *LRRK2 *families (Table 2). Comparatively, 8% of the affected parent-offspring-ascertained families were *LRRK2 *families and none of the families ascertained through more distantly related affected relative pairs included *LRRK2 *carriers.

The average ages of onset of PD in *LRRK2 *carriers and non-carriers are shown in Table 3. There were no significant differences in onset age between *LRRK2 *carriers and non-carriers, between carriers of different types of *LRRK2 *mutations, or between men and women.

**Table 3:** Mean onset ages stratified by *LRRK2 *mutations and sex are shown

	G2019S		R1441C		No *LRRK2 *mutations	
	Men	Women	Men	Women	Men	Women
No. of cases (%)	32 (53.3%)	28 (46.7%)	1 (25%)	3 (75%)	549 (56.9%)	416 (43.1%)
Mean onset age	60.0	59.9	53.0	67.0	60.0	59.7
No. of familial cases (%)	29 (53.7%)	25 (46.3%)	1 (25%)	3 (75%)	471 (55.7%)	374 (44.3%)
Mean onset age	59.9	59.3	53.0	67.0	60.9	59.9
No. of idiopathic cases (%)	3 (50%)	3 (50%)	0	0	78 (65.0%)	42 (35.0%)
Mean onset age	61.0	64.3	-	-	55.0	57.7

No significant differences in onset age were detected in any category.

Family history data from 22 of the 24 families carrying *LRRK2 *mutations and 340 of the 377 of non-*LRRK2 *families ascertained through the affected sibling pair method was available for estimation of penetrance of the *LRRK2 *mutations. Age-dependent penetrance was estimated in 22 of 24 families carrying *LRRK2 *mutations ascertained through the affected sibling pair method and separately in 20 of the 22 families carrying the G2019S data (two families had incomplete family history data). The two families carrying the R1441C mutation were not analyzed separately due to the small sample size. The age -dependent penetrance of PD among the parents of non-*LRRK2 *families ascertained by the affected sibling pair method was estimated to provide a baseline risk for comparison.

**Figure 1:**
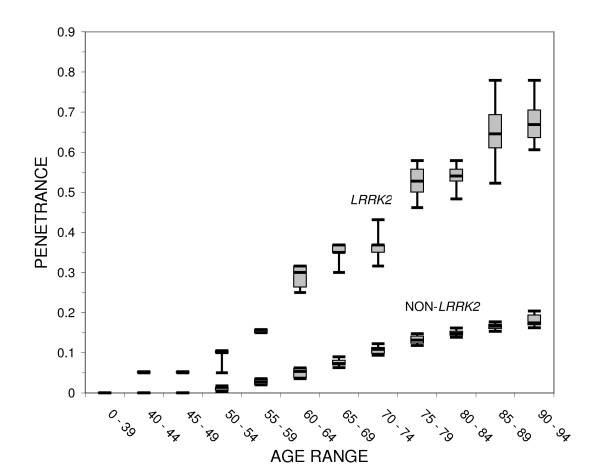
The box plot shows the median and 25^th ^and 75^th ^percentiles with whiskers extending to the 5^th ^and 95^th ^percentiles of the penetrance at each age range for G2019S carriers and for non-*LRRK2 *carriers among families recruited for at least two Parkinson's disease-affected siblings.

The median, quartiles and 5^th ^and 95^th ^percentile of the penetrance calculated in 1000 random selections of a single parent from each G2019S family are shown in Figure 1. In order to provide a baseline for comparison, the age-dependent risk of PD in parents of non-*LRRK2 *PD-affected siblings is also shown. In the age range of 50 to 54, the median estimated penetrance is 10% among the G2019S carriers, compared with 1% in non-*LRRK2 *carriers. At the maximum observed onset age range (90 to 94 years), the median estimated penetrance reaches 67% for the parents in G2019S mutation families, approximately four times greater than the expected risk in parents of non-*LRRK2 *PD-affected siblings. Including the R1441C families (data not shown) increases the maximum observed penetrance to 70%.

There was no observed difference in penetrance between fathers and mothers; none of the 1000 log-rank tests showed a significant difference in penetrance by sex (data not shown).

## Discussion

*LRRK2 *mutations were identified in both randomly ascertained idiopathic PD cases and in families with multiple PD-affected members. As expected, *LRRK2 *mutations were more common among families with multiple cases of PD than in randomly ascertained cases and more common in families recruited as parent-offspring pairs with an apparent dominant transmission than in those recruited as affected sibling pairs. The number of mutations identified in cases with no family or parental history of PD (4.8%) is consistent with previous reports of G2019S *LRRK2 *mutations.

The R1441C mutation was only observed in two sets of siblings, both of whom reported a parental history of PD and at least one other PD-affected relative. While this pattern of affection is suggestive of full penetrance for this mutation, the sample is too small to justify this inference with confidence. Future studies of a larger group of R1441C and other *LRRK2 *mutations may provide interesting insights into the mechanisms of *LRRK2*-related PD through comparison of the age-dependent penetrance among the different *LRRK2 *mutations and their modifiers.

The more common G2019S mutation shows reduced penetrance in this sample of familial PD. However the penetrance estimated here, in a sample composed entirely of familial PD, is substantially higher than that reported previously in a sample of randomly ascertained idiopathic PD cases (67% versus 33% at age 85 [11]). Conversely, studies of G2019S in large familial samples [4-7] report higher penetrance (70 to 100% at age 75) than the 67% at age 85 reported here. Contrasting penetrance in families selected to include multiple affected members (for example 'familial PD') with penetrance in families with a single affected member (for example 'sporadic' or 'idiopathic' PD) will, by definition, generate lower estimates in the latter group if the multiple affected members required for selection in the former group are included in the estimates. We sought to avoid this bias towards inflated penetrance by studying only the parents of subjects who were recruited as sibling pairs. Nonetheless, the comparison of estimates across these different study designs indicates variability in *LRRK2 *penetrance among families. This suggests the presence of other genetic factors which modify the actions of *LRRK2 *mutations and influence the penetrance. Mutations in *LRRK2 *have been associated with not only typical PD but with other pathologies as well [3]. The questionnaires used for this study do encompass other disorders but are most specifically designed to identify likely PD cases. Therefore these estimates should be considered only as estimates of the penetrance of typical PD and may underestimate the penetrance of any manifestation of *LRRK2*-related pathology.

A positive family history of PD is associated with increased risk of PD [13] and the 17% risk among parents of PD sibling pairs in non-*LRRK2 *families is higher than the risk expected in the general population (see Figure 1). Genetic factors which influence the penetrance of *LRRK2 *mutations may either interact only with that gene or may be factors which influence the susceptibility to familial PD among non-*LRRK2 *carriers as well. The presence of nine phenocopies identified among the relatives of G2019S carriers suggests that at least some genetic susceptibility factors are common to both *LRRK2 *and non-*LRRK2*-related PD.

No significant difference in penetrance was seen between fathers and mothers, although men are at a generally higher risk of PD than women [14]. This suggests the possibility that modifiers of *LRRK2 *penetrance are independent from the sex-dependent modifiers of PD risk in general. Similarly however, no significant difference in penetrance was seen between fathers and mothers of the affected sibling pairs of non-*LRRK2 *families, which is more surprising, given that this sample is predominantly male (see Table 3). These two findings suggest that sex-dependent modifiers of PD risk may have a reduced effect in both *LRRK2 *and non-*LRRK2*-related familial PD, relative to that seen in sporadic or idiopathic PD. This is consistent with a recent study in a large Ashkenazi sample which also suggested that the risk of *LRRK2*-related PD is similar in men and women as opposed to the risk of idiopathic PD which was increased in men [15].

## Conclusion

While the PD penetrance estimate of 67% for *LRRK2 *mutation families is four times that seen in non-*LRRK2*-related PD families, these studies suggest that there is a significant level of non-penetrance among *LRRK2 *carriers. Additionally, the presence of phenocopies and the increased penetrance in PD families compared with randomly ascertained PD samples suggest genetic susceptibility factors which affect risk of both *LRRK2*-related and idiopathic PD. Thus, identification of the factors which influence penetrance may hasten discovery of disease-modifying factors in both *LRRK2 *families as well as in idiopathic PD.

## Competing interests

The authors declare that they have no competing interests.

## Authors' contributions

JCL participated in the study design, recruited study subjects, performed the statistical analysis and drafted the manuscript. MS performed sequencing and reviewed the initial manuscript. MFL, OS, CK, LIG, MHM, JHG, GFW, RW, MG, BAR, JSP, AA, HAS, CS, SG, GP, MZ, MSM, KBB, II, IL, GN, AC, MN, ED, SI, DJB, PFC, PPP, JA, ATM, KO, SJS, RR, BS, JTS and FC recruited study subjects and reviewed the initial manuscript. AEH, JBW, JML, ALD, JFG participated in study design and reviewed the initial manuscript. SW and MWN performed genotyping and reviewed the initial manuscript. TM and KWH recruited study participants, participated in the coordination of the study and reviewed the initial manuscript. RHM participated in the design and coordination of the study and in drafting the manuscript. All authors read and approved the final manuscript.

## Pre-publication history

The pre-publication history for this paper can be accessed here:



## References

[B1] Mayeux R (2003). Epidemiology of neurodegeneration. Annual review of neuroscience.

[B2] Tan EK, Skipper LM (2007). Pathogenic mutations in Parkinson disease. Human mutation.

[B3] Singleton AB (2005). Altered alpha-synuclein homeostasis causing Parkinson's disease: the potential roles of dardarin. Trends in neurosciences.

[B4] Funayama M, Hasegawa K, Kowa H, Saito M, Tsuji S, Obata F (2002). A new locus for Parkinson's disease (PARK8) maps to chromosome 12p11.2-q13.1. Annals of neurology.

[B5] Paisan-Ruiz C, Jain S, Evans EW, Gilks WP, Simon J, Brug M van der, Lopez de Munain A, Aparicio S, Gil AM, Khan N, Johnson J, Martinez JR, Nicholl D, Carrera IM, Pena AS, de Silva R, Lees A, Marti-Masso JF, Perez-Tur J, Wood NW, Singleton AB (2004). Cloning of the gene containing mutations that cause PARK8-linked Parkinson's disease. Neuron.

[B6] Kachergus J, Mata IF, Hulihan M, Taylor JP, Lincoln S, Aasly J, Gibson JM, Ross OA, Lynch T, Wiley J, Payami H, Nutt J, Maraganore DM, Czyzewski K, Styczynska M, Wszolek ZK, Farrer MJ, Toft M (2005). Identification of a novel LRRK2 mutation linked to autosomal dominant parkinsonism: evidence of a common founder across European populations. American journal of human genetics.

[B7] Lesage S, Ibanez P, Lohmann E, Pollak P, Tison F, Tazir M, Leutenegger AL, Guimaraes J, Bonnet AM, Agid Y, Durr A, Brice A (2005). G2019S LRRK2 mutation in French and North African families with Parkinson's disease. Annals of neurology.

[B8] Ferreira JJ, Guedes LC, Rosa MM, Coelho M, van Doeselaar M, Schweiger D, Di Fonzo A, Oostra BA, Sampaio C, Bonifati V (2007). High prevalence of LRRK2 mutations in familial and sporadic Parkinson's disease in Portugal. Mov Disord.

[B9] Clark LN, Wang Y, Karlins E, Saito L, Mejia-Santana H, Harris J, Louis ED, Cote LJ, Andrews H, Fahn S, Waters C, Ford B, Frucht S, Ottman R, Marder K (2006). Frequency of LRRK2 mutations in early- and late-onset Parkinson disease. Neurology.

[B10] Ozelius LJ, Senthil G, Saunders-Pullman R, Ohmann E, Deligtisch A, Tagliati M, Hunt AL, Klein C, Henick B, Hailpern SM, Lipton RB, Soto-Valencia J, Risch N, Bressman SB (2006). LRRK2 G2019S as a cause of Parkinson's disease in Ashkenazi Jews. The New England journal of medicine.

[B11] Goldwurm S, Zini M, Mariani L, Tesei S, Miceli R, Sironi F, Clementi M, Bonifati V, Pezzoli G (2007). Evaluation of LRRK2 G2019S penetrance: relevance for genetic counseling in Parkinson disease. Neurology.

[B12] Marder K, Levy G, Louis ED, Mejia-Santana H, Cote L, Andrews H, Harris J, Waters C, Ford B, Frucht S, Fahn S, Ottman R (2003). Accuracy of family history data on Parkinson's disease. Neurology.

[B13] Rocca WA, McDonnell SK, Strain KJ, Bower JH, Ahlskog JE, Elbaz A, Schaid DJ, Maraganore DM (2004). Familial aggregation of Parkinson's disease: The Mayo Clinic family study. Annals of neurology.

[B14] Bower JH, Maraganore DM, McDonnell SK, Rocca WA (1999). Incidence and distribution of parkinsonism in Olmsted County, Minnesota, 1976–1990. Neurology.

[B15] Orr-Urtreger A, Shifrin C, Rozovski U, Rosner S, Bercovich D, Gurevich T, Yagev-More H, Bar-Shira A, Giladi N (2007). The LRRK2 G2019S mutation in Ashkenazi Jews with Parkinson disease: is there a gender effect?. Neurology.

